# Role of Cardiolipin in Mitochondrial Function and Dynamics in Health and Disease: Molecular and Pharmacological Aspects

**DOI:** 10.3390/cells8070728

**Published:** 2019-07-16

**Authors:** Giuseppe Paradies, Valeria Paradies, Francesca M. Ruggiero, Giuseppe Petrosillo

**Affiliations:** 1 Department of Biosciences, Biotechnologies and Biopharmaceutics, University of Bari, 70125 Bari, Italy; 2 Maasstad Ziekenhuis, 3079 DZ Rotterdam, The Netherlands; 3 Institute of Biomembranes, Bioenergetics and Molecular Biotechnologies (IBIOM), National Research Council (CNR), 70126 Bari, Italy

**Keywords:** Cardiolipin, mitochondrial structure-function, physiopathology, pharmacological agents

## Abstract

In eukaryotic cells, mitochondria are involved in a large array of metabolic and bioenergetic processes that are vital for cell survival. Phospholipids are the main building blocks of mitochondrial membranes. Cardiolipin (CL) is a unique phospholipid which is localized and synthesized in the inner mitochondrial membrane (IMM). It is now widely accepted that CL plays a central role in many reactions and processes involved in mitochondrial function and dynamics. Cardiolipin interacts with and is required for optimal activity of several IMM proteins, including the enzyme complexes of the electron transport chain (ETC) and ATP production and for their organization into supercomplexes. Moreover, CL plays an important role in mitochondrial membrane morphology, stability and dynamics, in mitochondrial biogenesis and protein import, in mitophagy, and in different mitochondrial steps of the apoptotic process. It is conceivable that abnormalities in CL content, composition and level of oxidation may negatively impact mitochondrial function and dynamics, with important implications in a variety of pathophysiological situations and diseases. In this review, we focus on the role played by CL in mitochondrial function and dynamics in health and diseases and on the potential of pharmacological modulation of CL through several agents in attenuating mitochondrial dysfunction.

## 1. Introduction

**Figure 1:**
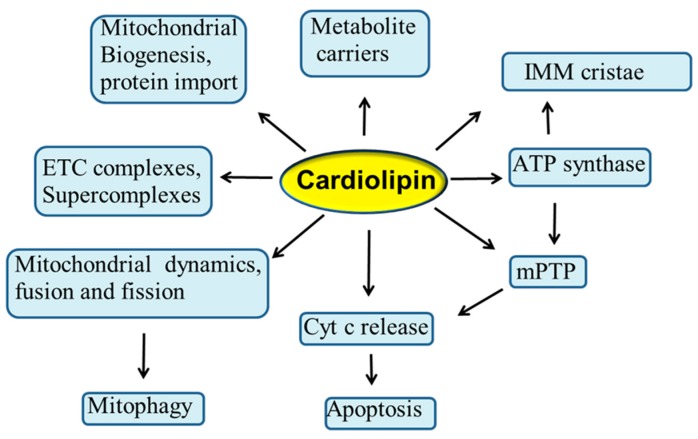
Proposed roles of cardiolipin in mitochondrial function and dynamics. Cyt c, cytochrome c; ETC, electron transport chain; IMM, inner mitochondrial membrane; mPTP, mitochondrial permeability transition pore. For details, see the text.

Mitochondria are considered to be the powerhouse of the cell, producing almost all energy required for cell metabolism through the process of oxidative phosphorylation (OXPHOS) [1]. These organelles are also involved in numerous other physiological processes in the cell, such as programmed cell death, autophagy, redox signaling, and Ca^2+^ homeostasis. Mitochondria contain two membranes: the outer mitochondrial membrane (OMM) and the inner mitochondrial membrane (IMM), while the compartment delimited by these two membranes is referred as intermembrane space. The cristae structures are tube-like invaginations of the IMM that project into the matrix and arbor the enzymatic complexes involved in OXPHOS process. Phospholipids are the main building blocks of mitochondrial membrane bilayers. OMM and IMM exhibit different lipid composition and an asymmetric distribution of phospholipids. These compounds are mainly synthesized in the endoplasmic reticulum (ER) and then transferred to the mitochondria. Phospholipids play a crucial role in the mitochondrial membrane’s architecture, function and dynamics and in the transport of proteins into the mitochondria [2,3]. Alterations in the phospholipid composition can affect the mitochondrial membrane integrity, permeability and fluidity, and hence the stability and activity of many IMM-associated proteins, including those involved in ETC and OXPHOS processes, with important implications in a variety of human diseases [4]. Cardiolipin (CL) is the signature phospholipid of energy-transducing membranes, including the IMM, where it constitutes approximately 15–20% of the total mitochondrial phosholipids. CL is unusual among all phospholipid species in that it exhibits a dimeric structure with four acyl chains and two phosphatidyl moieties that are linked to the glycerol. This unique structure of CL yields a conical shape that is the origin of its curvature sensing abilities [5,6,7]. Accumulating evidence suggests that CL plays a central role in several reactions and processes of the mitochondrial metabolism, including respiration and energy production [5,8,9,10,11]. In addition, this phospholipid is involved in mitochondrial cristae morphology and stability [5,12], in mitochondrial quality control and dynamics through fission and fusion [10,13,14], in mitochondrial biogenesis and protein import [15,16], in mitophagy [17,18], as well as in several mitochondrial steps of the apoptotic process, serving as a binding platform to recruit apoptotic factors [19,20,21] (Figure 1). Due to its high content of unsaturated fatty acids and its location in the IMM near to ETC complexes, the main sites of reactive oxygen species (ROS) production, CL is particularly prone to peroxidation, an event that may affect many CL-dependent reactions and processes [9,22,23,24]. In addition, the accumulation of oxidized CL in the OMM serves as important signaling platform during the apoptotic process, resulting in the opening of the mitochondrial permeability transition pore (mPTP) and in the release of cytochrome c (cyt c) from mitochondria to the cytosol [19,20,25,26] (Figure 1). It is conceivable that alterations occurring in the CL profile may negatively impact the activity of a variety of mitochondrial proteins and enzymes, including the ETC and OXPHOS complexes, thus compromising mitochondrial function and dynamics. These events may play a causative role in the etiology and progression of several pathophysiological situations and diseases, including Barth syndrome, myocardial ischemia/reperfusion injury, heart failure, diabetes and neurodegenerative disorders [23,27,28,29,30,31,32,33]. Thus, understanding the role played by CL in the mitochondria, will shed light on the molecular mechanisms involved in the regulation of mitochondrial function in health and diseases.

In the present review, we summarize the most recent advances in the study of the role played by CL in mitochondrial function and dynamics and in cell death, in health and disease. Pharmacological strategies able to prevent or attenuate mitochondrial dysfunction targeting directly or indirectly CL and their implications in pathophysiology, are also examined.

## 2. Cardiolipin Biosynthesis and Remodeling

Unlike the other phospholipids, CL is synthesized almost exclusively within the mitochondrion to yield a nascent form of this phospholipid with non-uniform acyl chains composition [5]. The synthesis of CL initiates from phosphatidylglicerol (PG) that is transported from the endoplasmic reticulum (ER) to the IMM. In the inner membrane, PG is converted to CL via a sequence of enzymatic reactions, the last one being catalyzed by the enzyme cardiolipin synthase using cytidin diphosphate-diacyl glycerol as substrate [5,34]. Following the de novo synthesis of CL on the matrix side of IMM, acyl chain remodeling is responsible for the final molecular composition of mature CL containing predominantly unsaturated fatty acids. Three different enzymatic pathways are involved in CL remodeling through the sequential action of CL-specific deacylase and transacylase [5,34] In one of these pathways, CL is first deacylated to monolyso-cardiolipin (MLCL) by CL-specific phospholipases and then the coenzyme-A independent acyltransferase Tafazzin mediates the reacylation to form mature CL [5,34,35]. The mutations in the *TAZ* gene encoding for Tafazzin cause Barth syndrome, a rare X-linked genetic disease [36,37]. Following de novo synthesis and remodeling, mature CL is translocated and assembled in the IMM and OMM. The translocation of CL is mediated by three different enzymes, phospholipase scramblase (PLS3), mitochondrial creatine kinase (mtCK), and nucleoside dophosphate kinase (NDPK) [38,39]. The activity of these kinases is modulated by CL and requires proteins aggregation which promotes CL clusters and CL membrane domain formation [40]. These CL domains are supposed to play an important role in the regulation of mitochondrial membrane structure and morphology and the clustering of proteins [40].

## 3. Cardiolipin and Mitochondrial Bioenergetics

### 3.1. Interaction of CL with Mitochondrial Substrate Carriers

The primary function of mitochondria is the production of energy through the OXPHOS process [1]. In addition to this role, mitochondria are involved in other vital metabolic processes in the cell, including citric acid cycle, fatty acid oxidation, the synthesis and degradation of amino acids, and the synthesis of iron-sulfur clusters and heme. To use these metabolic pathways, metabolites have to be continuously translocated and exchanged between the mitochondrial matrix space and the cytosol. The IMM contains a mitochondrial carrier family that mediates the translocation of several metabolites between intermembrane and matrix space [41]. Mutations in certain members of this mitochondrial carrier family are involved in several human diseases [42]. Cardiolipin has been shown to interact with and to be required for optimal activity of several mitochondrial carrier proteins, including ADP/ATP carrier (ANT), phosphate carrier (PiC), pyruvate carrier, tricarboxylate carrier and the carnitine/acylcarnitine translocase [9,43]. The ANT plays an important role in energy metabolism by allowing the ATP formed by OXPHOS to be transferred from the IMM to intermembrane space. The activity of the ANT has been shown to be optimal only in the presence of tetralinoleoyl-CL whereas, other CL species and also other phospholipids were ineffective in catalyzing the ANT activity [43]. The crystal structure of ANT indicates that there are two or three molecules of CL tightly bound for monomer of this protein [44]. These tightly bound CL molecules are supposed to be involved in linking the monomers within the dimer and in stabilizing the dimeric structure of ANT, which is the active form of this protein [44].

### 3.2. Interaction of CL with ETC Complexes

It has been well established that CL plays a central role in several mitochondrial bioenergetic processes [5,8,9,10,11]. In fact, this phospholipid has been shown to interact with several IMM proteins and enzymes, including, among others, enzyme complexes involved in the ETC and OXPHOS processes. The respiratory chain consists of four complexes (I –IV), which catalyze the electron transport from NADH or FADH_2_ to molecular oxygen. The transmembrane proton gradient created by the electron transfer across the IMM, serves as an energy source for the production of ATP by complex V (ATP synthase). Cardiolipin interacts with all the ETC complexes and it is required for their structural integrity and proper enzymatic activity. Specific binding sites for CL in complexes I, III and IV have been detected [11,45]. The detergent-solubilized complex III and IV contain several bound CLs, which are required for their stability and functional activity [46]. Removal of these bound CLs leads to almost complete loss of activity and dissociation of subunits of these complexes, while re-association of CL results in the stabilization of the quaternary structure and restoration of the full activity. This indicates that CL is essential for catalytic function of complexes III and IV acting as an allosteric ligand that stabilizes the proper active conformation of these enzyme complexes. Tightly bound CL molecules have been detected in the crystal structures of ETC protein complexes [11,47]. Two CLs within complex IV, one CL within complex III and complex II and four CLs within complex I have been resolved in each of their crystal structure [11]. These CL molecules are buried in crevices between transmembrane helices, and are bound to specific sites of the proteins, possibly stabilizing the association of specific membrane spanning subunits. These data indicate that CL is an integral component of these proteins and it is required for their proper folding and optimal functioning. In addition, an active role for CL in proton translocation in complex III and IV has been proposed [45].

### 3.3. Role of CL in Supercomplex Formation

Several studies indicate that ETC complexes in the IMM are organized in higher order structures, referred generically as supercomplexes or “respirasomes”, rather than existing as individual complexes [48,49]. Different models of supercomplexes, involving components of the electron transport chain (complexes I, III and IV), complex V and ANT, have been proposed [48,49,50]. For example, in mammalian mitochondria, the existence of a supercomplex composed of complex I, complex III dimers and several monomers of complex IV has been reported [51]. This supercomplex organization of the ETC provides structural/functional linkages between the respiratory complexes resulting in a more efficient electron transfer, thereby preventing excessive ROS generation. CL is specifically required for association, stabilization and functioning of individual complexes into supercomplexes [52,53].

As mentioned above, CL is able to bind and interact with a variety of structurally unrelated proteins. The binding of CL to the protein may involve strong hydrophilic interactions, such as electrostatic forces, hydrogen bonds and water molecules, between the polar head group of CL with several amino acid residues of the protein. Essential in the interaction between CL and protein is the ability of this phospholipid to insert, with its negatively charged hydrophilic domain, into the cavities and grooves between hydrophobic interface of the protein, while providing specific ionic bridges at the water hydrophobic interface.

## 4. ROS and Cardiolipin Oxidation

ROS are considered an important contributing factor in the etiology and progression of several diseases, although this view has been challenged, in part, based on the failure of some anti-oxidants to protect against several pathophysiological situations characterized by enhanced oxidative stress [54]. Mitochondria are considered a major source of ROS which are most likely generated during the electron transfer to molecular oxygen via ETC [55]. The rapid movement of electrons through the ETC can result in the leakage of electrons that can form O_2_^•−^. Complexes I and III, are considered the major sites of O_2_^•−^ generation [55]. Due to their high content of unsaturated acyl chain and their location in the IMM near to the locus of ROS production, CL molecules may readily undergo oxidative attack by these radical species. Oxidation of CL may affect several CL-dependent reactions involved in mitochondrial bioenergetics. Our in vitro studies on mitochondria isolated from different tissues have shown that oxidation of CL affects the activity of Complexes I, III and IV, while the addition of CL-containing liposomes restores the normal activity of these complexes [56,57,58]. In addition, oxidized CL and its externalization on the OMM are involved in several steps of the apoptotic process, such as mPTP opening and cytochrome c release from mitochondria [26]. Thus, oxidation of cardiolipin may underlie mitochondrial dysfunction in several pathophysiological situations and may represent and important target for antioxidant defense strategies. Recent studies have provided evidence for the formation of 4-hydroxy-trans-2-nonenal (HNE) from CL oxidation via cross-chain peroxyl radical addition and decomposition under oxidative stress conditions [59]. This finding may have important implications in the apoptotic process and other biological activities of HNE. In fact, this compound is considered one of the most important signaling molecules, being involved in a variety of signaling events in a physiological context, including inhibition of DNA synthesis and inactivation of proteins and enzyme [60].

## 5. Interaction of Cardiolipin with ATP-Synthase and Implications for mPTP

Mitochondrial ATP-synthase is a multimeric enzyme complex consisting of two functional domains, F_o_ situated in IMM and F_1_ located in mitochondrial matrix. This enzyme complex utilizes the energy created by the proton electrochemical gradient to synthesize ATP and this is accomplished via the rotation of the central stalk and a ring of c subunits of the ATP synthase in the membrane domain. The ATP-synthase can exist as a monomeric inactive and a dimeric active form. The dimeric form of this enzyme has been localized at the level of the IMM cristae tip [61,62]. During the OXPHOS process catalyzed by ATP synthase, large amounts of protons are transferred from one side to the other side of the IMM, causing large pH changes. CL is supposed to act, with its phosphate head group domain, as a proton trap within the IMM, thus minimizing pH changes in the intermembrane space and supplying protons for the ATP synthase activity [63]. Moreover, ATP-synthase has been shown to form higher oligomeric assemblies that consist of rows of dimers essential for cristae formation and stabilization [61,62]. CL appears to be critical for the oligomerization and order in these ATP-synthase assemblies, which are likely to affect cristae morphology and hence, energy production [64].

**Figure 2:**
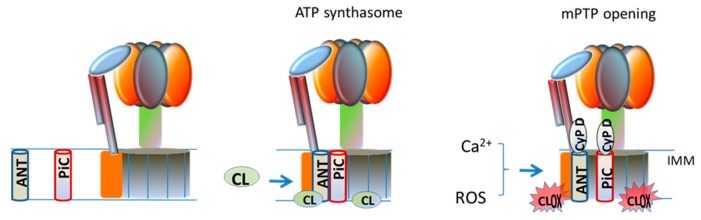
Proposed role for cardiolipin in ATP synthasome formation and mPTP opening. ANT, adenine nucleotide translocator; CL, cardiolipin; CLox, oxidized cardiolipin; Cyp D, cyclophilin D; IMM, inner mitochondrial membrane; mPTP, mitochondrial permeability transition pore; PiC, phosphate carrier. For details, see the text.

mPTP is a high conductance channel that generates a sudden increase in the IMM permeability to ions and small solutes, causing collapse of membrane potential and uncoupling of OXPHOS [65]. Potential candidates in the formation and modulation of the mPTP are, cyclophilin D (CypD) in the matrix, ANT and PiC in the IMM and VDAC (voltage dependent anion channel) in the OMM [66,67]. Recent studies have demonstrated that ATP-synthase is another putative component of mPTP within the IMM [66,67,68]. CL has been shown to interact with- and to be required for the stabilization and functioning of the ATP-synthase facilitating its rotation supported by the transmembrane proton gradient [69,70]. The involvement of ATP-synthase in mPTP formation is supported by the finding that CypD comigrates with mitochondrial F_0_F_1_ ATP synthase in blue native gel and the subunit OSCP as a binding site [71]. Two hypotheses about the involvement of ATP-synthase in the formation of mPTP have been proposed. The first proposal suggests that the pore forms at the monomer–monomer interface of the dimer of ATP-synthase [71], while the second proposal focuses on the involvement of the c-subunit ring of theF_o_in mPTP formation [71]. The ATP-synthase, PiC and ANT form a large supercomplex in mitochondria, called ATP synthasome [52] Although the primary role of this supercomplex is that of improving the efficiency of the OXPHOS process, an involvement of ATP synthasome in the formation/regulation of mPTP, has been also suggested [72,73]. The oligomerization of the ATP-synthasome seems to be modulated by CL molecules [74], which interact with ATP synthase, ANT and PiC, all components of this supercomplex, probably gluing these proteins together. Ca^2+^ ions, which are the main inducers of mPTP opening, exhibit high affinity for CL. It has been suggested that, under conditions promoting mPTP opening, the binding of Ca^2+^ ions to annular CL at the interface between the PiC, ANT and ATP synthase, would cause conformational changes in the ATP synthasome, promoting mPTP formation (Figure 2) [75]. It is reasonable to hypothesize that, in the presence of Ca^2+^, abnormalities in the content and composition of CL and, notably its oxidation, may perturb the interactions between the protein components of the ATP synthasome, inducing destabilization in this supercomplex, thus promoting mPTP opening. This hypothesis is also supported by our in vitro studies carried out on isolated rat heart mitochondria, showing that addition of oxidized CL to these organelles induces in the presence of Ca^2+^ ions, mPTP opening [26].

## 6. Interaction of CL with Cytochrome C and Apoptosis

Cytochrome c is a nuclear-encoded mitochondrial protein which plays multiple important roles in mitochondrial function [76]. The primary role of this protein is that of transferring electrons in the ETC and thus, it is indispensable part of the OXPHOS process. Cytochrome c is also supposed to act as a radical scavenger within the mitochondria by removing unpaired electrons from superoxide, thus regenerating oxygen [77]. Another important role of this hemeprotein in mitochondria is its involvement in several steps of the apoptotic process. In fact, the release of cyt c from mitochondria to the cytosol is an essential event in the induction of the apoptotic cascade that ultimately leads to programmed cell death [19,76]. Cyt c interacts with CL through two separate binding sites [78]. One site likely involves electrostatic interactions between the phosphate head group of CL and lysine residues of cyt c and participates in the electron transfer and radical scavenging function of this hemeprotein. The second site involves hydrophobic interactions between one of the unsaturated acyl chain of CL and an aspartic acid residue of cyt c [79]. During apoptosis, a redistribution of CL occurs from the IMM to the OMM. Externalized CL interacts with cyt c forming a cyt c/CL complex which exhibits a peroxidase activity capable of catalyzing oxygen-dependent peroxidation of substrates [80]. Cardiolipin promotes the peroxidase activity of cyt c by destabilizing the tertiary structure of this hemeprotein through the hydrophobic interactions. The peroxidase activity of the CL-cyt c complex targets the unsaturated acyl chains of CL, resulting in an increase of oxidized CL [81]. Cyt c exhibits a lower affinity toward oxidized CL and this facilitates its detachment from the IMM and its release from mitochondria to cytosol during apoptosis [19,82]. CL is involved in other steps of the apoptotic program, providing an activating platform in OMM for caspase 8, which is processed and translocated to mitochondria upon Fas receptor activation [20,83]. Activation of caspase 8 results in the cleavage of the proapoptotic protein Bid to the trunked form, t-Bid, which translocates to the OMM, triggering the oligomerization of the proteins Bax and Bak, thus leading to OMM permeabilization [20,83].

## 7. Role of Cardiolipin in Mitochondrial Dynamics

In addition to their primary role in energy production through the OXPHOS process, mitochondria are involved also in a variety of other vital metabolic processes to preserve intracellular homeostasis. Mitochondria are dynamic organelles that constantly undergo fusion and division processes to maintain their proper morphology essential for their normal functions. Thus, it is important to have strict quality control mechanisms to ensure healthy mitochondrial network for cell fate. Quality control mechanisms are mainly regulated by mitochondrial dynamics and mitochondria-specific autophagy, known as mitophagy, through processes such as mitochondrial fission and fusion, which facilitates the equilibration of mitochondrial components, including DNA, proteins and metabolites [84]. Mitochondria dynamics are regulated by the balance of expression levels between fission and fusion proteins. Mitochondrial fission is a multistep process characterized by the division of one mitochondrion into daughter mitochondria and it is mediated by the large cytosolic GTPase, Dynamin-related protein1 (Drp1) [85]. During mitochondrial division, Drp1 is recruited to the OMM, where it forms a ring-like structure around mitochondria [86]. Mitochondrial fusion is the union of two mitochondria, resulting in one mitochondrion. This process is mediated by membrane-anchored proteins, mitofusin (Mfn)-1,2 and optic atrophy (OPA)-1, which promote the fusion of OMM and IMM, respectively [13,85]. The regulation of mitochondrial fission and fusion processes results in either a large number of small, round-shaped mitochondria, or elongated and highly connected mitochondria. The balance between these two processes is required to ensure proper mitochondrial function and also to respond to metabolic state of the cell [85]. The deficiency of fusion and fission proteins leads to the fragmentation of mitochondria. The mitochondrial fusion-division dynamics are also important to modulate CL abundance [87]. The process of mitophagy is responsible for the degradation and recycling of injured, dysfunctional mitochondria [85]. Recent in vitro and in vivo studies have shown that CL is involved in several steps of mitochondrial dynamics and morphology, including fusion and fission [6,10,13,14,85], mitophagy [88,89] and protein insertion and assembly into the mitochondria [15,16]. The unique biochemical and biophysical properties of CL may account for its involvement in mitochondrial dynamics, notably fusion and fission processes. CL is a dimeric phospholipid with a small anionic head group, relative to the four unsaturated acyl chains, predominantly linoleoyl acyl chains, which render this phospholipid effectively cone shaped in the membrane phospholipid bilayer. Therefore, CL exhibits complex phase behavior in the IMM with propensity to form and stabilize negative membrane curvatures and to transition from a lamellar, bilayer phase to the non-lamellar inverted hexagonal (H_II_) configuration. These biochemical and biophysical properties of CL may be relevant to phospholipid changes which occur during mitochondrial fission and fusion and other processes of mitochondrial dynamics. Indeed, the involvement of Drp1 in promoting mitochondrial fission is critically dependent on its ability to interact with CL [90]. It has been proposed that Drp1 intercalates into the hydrocarbon core of the mitochondrial membrane bilayer where specifically associates with CL, restricting its motion and inducing transient CL non-bilayer transition that promotes membrane constriction [13,90]. Drp1 and CL function cooperatively in facilitating membrane remodeling and fission during mitochondrial division [90]. CL plays important roles in mitochondrial fusion through the biogenesis and assembly of OPA1, that mediates mitochondrial membranes fusion [14,91]. In mammals, OPA1 localization in only one of the two opposing mitochondria is sufficient to promote the fusion of both mitochondrial membranes. Incubation of OPA1 with reconstituted CL-containing liposomes results in a heterotypic interaction between OPA1 and CL, promoting membrane fusion [14], whereas no membrane fusion occurs when liposomes either lack OPA1 or contain low amount of CL. These results suggest that the amount of CL is critical for mitochondrial membranes fusion. In addition, the length and the level of saturation of CL acyl chains appear to influence mitochondrial fusion, with longer length and higher unsaturation enhancing this process [14].

### CL in Mitophagy

Mitophagy refers to the selective degradation and removal of superfluous, damaged and dysfunctional mitochondria by autophagy [92]. During mitophagy, injured mitochondria are first incorporated into a double membrane structure, the phagofores, forming the autophagosomes and then, fuses with lysosomes to target their content for degradation [93]. CL is believed to influence mitochondrial quality control process through the modulation of mitophagy. Recent studies have shown that mitochondrial injury, depolarization and other damage signals cause the migration of CL from the IMM to the OMM, where it is exposed to the cytosol [94]. This externalized CL appears to have important signaling function during mitophagy, forming an essential platform on the surface of the OMM where CL binds to specific sites of light chain 3 (LC3), a homolog of autophagy-related gene 8 (Atg8) in yeast. The binding CL-LC3 contributes to recognition of damaged and dysfunctional mitochondria, and initiating autophagosome formation, leading ultimately to the development of mitophagy [94]. An involvement of CL in mitophagy, is also supported by the finding that a conserved domain of Beclin 1, a mammalian ortholog of Atg 6 in yeast and another member and central regulator of the autophagic machinery, directly binds to CL on the OMM, to induce or inhibit mitophagy [95]. Dysregulation of the mitochondrial quality control machinery and defects in mitochondrial function may contribute to pathogenesis and development of several diseases.

## 8. CL Alterations and Diseases

**Figure 3:**
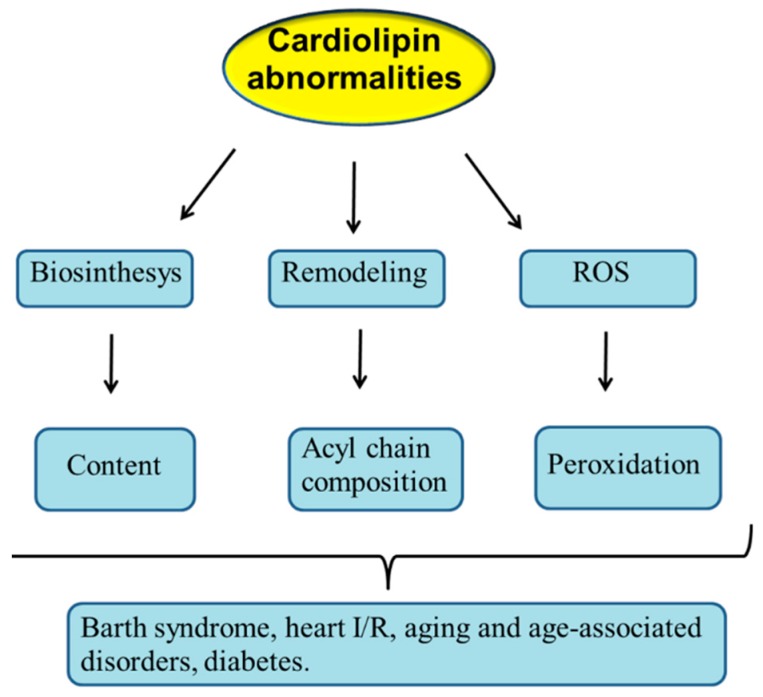
Cardiolipin abnormalities in diseases. I/R, ischemia/reperfusion, ROS, reactive oxygen species. For details, see the text.

As described above, CL is involved in the regulation of mitochondrial function and dynamics. Thus, it can be predicted that abnormalities occurring in CL structure, content and/or acyl chains composition may result in mitochondrial dysfunction with important implications in mitochondrial pathophysiology. Abnormalities in mitochondrial CL profile may occur as a consequence of dysregulation in its biosynthesis and remodeling and also following its oxidation by ROS attack (Figure 3).

### 8.1. Barth Syndrome

Barth syndrome (BTHS) is a rare X-linked genetic disorder characterized by a broad spectrum of clinical manifestations, including cardiomyopathy, skeletal muscle weakness, neutropenia, organic aciduria and growth retardation [40,96]. This rare disease is caused by mutations in the *TAZ* gene which encodes tafazzin, a phospholipid transacylase that promotes CL acyl chain remodeling [97]. BTHS is the first genetic disease known in which a clear association between the pathological phenotype and alterations in CL profile has been established. Absence of tafazzin or defect in its activity result in altered mitochondrial CL profiles in a variety of tissues, such as an increased levels of MLCL and a lower CL content [35]. The increase in the MLCL-to-CL ratio is a very specific and sensitive biomarker of BTHS and it is often utilized in diagnosis of this rare disease in patients. As described above, CL is required for optimal activity of respiratory chain complexes, and for their assembly and stabilization into supercomplexes, and this function cannot be replaced by other phospholipids, including MLCL [53]. Consistent with its altered CL profile, the activity of the ETC complexes is decreased in BTHS [37]. It has been also reported that ETC supercomplexes are destabilized in CL-depleted heart mitochondria from BTHS Tafazzin knockdown mouse, resulting in abnormal energy metabolism, impairment of the ETC activity and an increase in ROS generation which, in turn, exacerbates mitochondrial dysfunction [98,99,100]. In addition to its involvement in ETC function, CL, due to its cone-shaped structure, segregates to domains of negative membrane curvature, contributing to the formation of IMM cristae and to the maintenance of their morphology and stability [12]. In BTHS, altered CL remodeling correlates with profound changes in cristae ultrastructure [101]. Thus, tafazzin deficiency, by altering CL acyl chain remodeling, leads to changes in CL profile which result in aberrant mitochondrial cristae morphology and ultrastructure. Given that mitochondrial cristae harbor the ATP synthase, perturbations in their ultrastructure and morphology may have a negative impact on the function of this enzyme complex and hence, on energy production. Consistent with this, BTHS mitochondria exhibit a decreased membrane potential and respiratory coupling index and increased proton leak [37], which may contribute to the observed mitochondrial bioenergetic decline in BTHS.

### 8.2. Myocardial Ischemia/Reperfusion Injury

Myocardial ischemia occurs as a consequence of obstruction of the blood flow through the myocardium resulting in loss of contractile function and myocardial damage. Reperfusion of the ischemic heart results in the induction of oxidative stress, cardiomyocyte death and subsequent irreversible myocardial injury, a phenomenon commonly called ischemia reperfusion (IR) injury [102]. This pathological condition is characterized by cardiomyocyte hypercontracture, abnormal left ventricular pressure, augmented vascular resistance, arrhythmia and elevated incidence of ventricular fibrillation. Heart tissue consumes large amounts of energy to support contractile activity. It is well documented that mitochondrial dysfunction and oxidative stress are deeply involved in myocardial I/R injury [103,104]. The major role of mitochondria in the heart is the production of ATP through the ETC and OXPHOS processes. Approximately 90% of cellular ATP produced by cardiomyocyte is utilized to support the contraction-relaxation cycle within the myocardium. Accumulating evidence indicate that ROS, which are mainly produced during mitochondrial respiration. play a central role in myocardial I/R injury [105,106,107]. ROS-induced oxidative damage is observed especially at the level of ETC complexes and membrane phospholipids [30,31,32]. A loss in the CL abundance and an increase in CL oxidation have been reported in rat heart mitochondria during ischemia and additional CL loss upon reperfusion [30,107]. The losses in CL levels are associated with parallel increases in the levels of oxidized CL. These changes in the CL profile appear to be responsible, in part, for the alterations in bioenergetics parameters, such as decreased rate of O_2_ consumption, lower activity of complexes I, III and IV and increased basal rate of H_2_O_2_, observed in the rat heart I/R model [32]. Of note, the addition of exogenous CL-containing liposomes to I/R rat heart mitochondria almost completely restored the activity of ETC complexes to control preischemic levels [108]. Collectively, these results suggest that the alterations in the activity of mitochondrial ETC complexes in myocardial I/R can be ascribed, at least in part, to CL abnormalities. A vicious cycle appears to be formed during heart I/R in which ROS-induced damage to CL leads to impairment of ETC complexes activity that, in turn, results in more ROS production, amplifying mitochondrial dysfunction and myocardial injury. Due to CL requirement for the assembly and stabilization of ETC complexes into supercomplexes, it is reasonable to hypothesize that alterations in mitochondrial CL profile, as those occurring during heart I/R, may result in defects in formation and stabilization of ETC supercomplexes. Consistent with this, a destabilization and dysfunction of ETC supercomplexes (I, III and IV) in rat heart mitochondria after sustained reperfusion, have been recently reported [30].

It is well documented that mPTP plays an important role in cardiomyocytes death occurring during myocardial I/R [72]. It has been shown that mPTP remains closed during ischemia, but rapidly opens following heart reperfusion [72]. Numerous compounds and factors are involved in the activation the mPTP opening, the most effective being the Ca^2+^ ions. Our in vitro studies on isolated rat heart mitochondria, have shown that micromolar concentrations of exogenously added oxidized CL to these organelles lowers the threshold of Ca^2+^ for inducing mPTP activation [26]. These results suggest that both Ca^2+^ ions and oxidized CL may play a coordinate role in mPTP opening, probably by interacting with the supercomplex ATP synthasome. Given that the mitochondrial levels of Ca^2+^ and oxidized CL increase upon heart reperfusion, it is hypothesized that both these two factors, by interacting with ATP synthasome, may induce conformational changes in this supercomplex, thus favoring mPTP opening. The induction of mPTP opening by oxidized CL and Ca^2+^ promotes the release of cyt c from mitochondria which leads ultimately to cardiomyocytes death. Changes in CL profile may also play a role in mitochondrial dynamics during heart I/R. As described in a previous section, the involvement of the Drp1 in promoting mitochondrial fission is dependent on CL interaction [90]. Abnormalities in CL profile may perturb the interaction between CL and Drp1, thus affecting mitochondrial fission and dynamics. During heart reperfusion an increase in mitochondrial fission, while a decrease in mitochondrial fusion have been reported [109]. Increased mitochondrial fusion is associated with an enhanced susceptibility to mPTP opening, release of cyt c and subsequent activation of the apoptotic process, resulting in cardiomyocyte death at the time of reperfusion [109]. All these events may have an important role in the etiology of mitochondrial dysfunction in myocardial I/R injury and may provide an important therapeutic target for treating this pathological condition.

### 8.3. Diabetes

Diabetes, one of the most common endocrine disease, is characterized by peripheral insulin resistance and pancreatic islet B-cell failure. This form of diabetes is commonly known as type II or diabetes mellitus (T2DM). A large body of experimental evidence indicate that oxidative stress and mitochondrial dysfunction are important contributing factors to pancreatic B-cell failure in the progression of T2DM [100,101,102,103,104,105,106,107,108,109,110,111,112]. ROS produced as result of metabolic stress, are mainly responsible for the structural and functional alterations observed in pancreatic β-cell mitochondria, resulting in impaired energy production. CL molecules are the main target of ROS attack due their high content of polyunsaturated fatty acids. Changes in CL content, and its fatty acids composition have been observed in animal model of diabetes [28]. Studies carried out on heart mitochondria isolated from streptozotocin (STZ)-treated diabetic animals, have shown a decrease in CL levels and abnormalities in the CL acyl chain remodeling, resulting in increased incorporation of the highly unsaturated fatty acids, including docosahexaenoic (DHA) and arachidonic (AA) acids [28]. This aberrant CL remodeling contributes to mitochondrial dysfunction in the heart of diabetic animals. ALCAT1 is an acyltransferase that catalyzes resynthesis of CL from lysocardiolipin, a key step involved in CL remodeling. This process plays an important role in inducing oxidative stress by promoting the remodeling of CL with long acyl chains, such as, DHA and AA, which are very enriched with double bonds, rendering CL particularly sensitive to ROS attack [113]. ALCAT1 has been shown to be upregulated under oxidative stress conditions as those occurring in diabetes and diet-induced obesity, resulting in an increase in CL species enriched with long chain unsaturated fatty acids that are highly susceptible to peroxidation, thus promoting further ROS production, mitochondrial dysfunction and insulin resistance [113,114]. CL is implicated in the modulation of mitochondrial dynamic interacting with several proteins involved in this process, including mitofusins, Drp1 and OPA1. Mitochondrial dynamic changes are closed related with insulin resistance. An increase in mitochondrial fission was observed in the skeletal muscle of obese rodents and humans which is associated with insulin resistance [115]. Rats fed a high-fat diet exhibit an enhanced mitochondrial fission and insulin resistance [116]. These findings suggest that CL may be involved in the regulation of insulin resistance through modulation of mitochondrial dynamics.

### 8.4. Parkinson’s Disease

Parkinson’s disease (PD) is one of the most common neurodegenerative disease, characterized by a progressive loss of dopaminergic neurons from nigrostriatal pathway, formation of Lewy bodies and microgliosis [117]. Patients affected by PD exhibit many symptoms, including impaired functions of motor skills, tremor, rigidity, sleep disorders and impaired cognitive process [118,119]. Oxidative stress, mitochondrial dysfunction and defective mitophagy are all considered important factors in the pathogenesis and progression of PD [118]. A primary contributing factor in the pathogenesis of PD is the altered aggregation of the α-synuclein (αS) protein into higher-order oligomers that play a central role in neuronal dysfunction and degeneration [120]. Neurotoxicity of αS oligomers is linked to their ability to interact with biological membranes, thereby affecting their integrity and function. The interaction between αS and CL may represent an important event through which cytosolic αS physically associate with mitochondrial membranes, altering their integrity, morphology and function [120]. Rat brain mitochondria or neuronal cell lines treated with prefibrillar αS oligomers exhibit dramatic alterations in their morphology and function, such as mitochondrial swelling, loss of mitochondrial membrane potential, release of cyt c, increased ROS production and enhanced levels of Ca^2+^. All these events result in mitochondrial bioenergetics defects and decrease in cell viability [120]. In addition, the association of αS oligomers with CL may destabilize the respiratory supercomplexes, with subsequent impairment of electron transfer, loss in energy production by OXPHOS and ROS overproduction [121,122]. Thus, a feed-forward cycle of enhanced ROS production, reduced respiratory chain activity and loss of energy production would be set up. Consistent with this concept, a loss in CL abundance associated with aberrant mitochondrial bioenergetics in neurons overexpressing the N-terminal domain of αS has been observed [120]. In addition, α-synuclein knockout mice were shown to exhibit a loss in both CL content and mitochondrial complex I/III activity [121]. It has also been reported that αS oligomers bound to CL form a large complex with cyt c, thereby acting as substrates for peroxidase activity of this hemeprotein, thus promoting the permeabilization of mitochondrial membrane and also contributing to oxidative stress in dopaminergic neurons [123]. Impairment of mitochondrial complex I activity is implicated in neurotoxicity and apoptosis in 1-methyl-4-phenyl-1,2,4,6-tetra-hydropyridine (MPTP) treated mice, an experimental animal model of PD [124]. In brain aging, oxidative stress and CL oxidation affect the mitochondrial complex I activity suggesting that both these two factors may be implicated in the pathogenesis of PD [125]. CL is also required for mitophagy and abnormalities in this phospholipid appear to be involved in the pathogenesis of PD as evidenced in human PD patients with mutations in Parkin and PINK1 genes, two important regulators of the mitophagy [126]. PINK1 mutations result in lower levels of CL in mitochondria, associated with oxidative stress and aberrant mitochondrial function [126,127]. Very recently, an involvement of ALCAT1, an enzyme that catalyzes CL remodeling, in mitochondrial dysfunction associated with PD, was suggested [128]. In a mouse model of PD, MPTP treatment resulted in oxidative stress and mitochondrial dysfunction in midbrain. Ablation of ALCAT1 gene or inhibition of ALCAT1 activity by pharmacological agents, prevented MPTP-induced neurotoxicity, mitochondrial dysfunction, apoptosis, and locomotive defects, typical of PD. In addition, pharmacological inhibition of ALCAT1 activity, improved mitophagy by promoting the recruitment of Parkin to dysfunctional mitochondria [128]. These results point to the involvement of ALCAT1 in the etiology of mouse model of PD and identify this enzyme as a potential drug target for treatment of this neurodegenerative disorder.

## 9. Cardiolipin Targeting Therapeutic Agents in Disease

**Figure 4:**
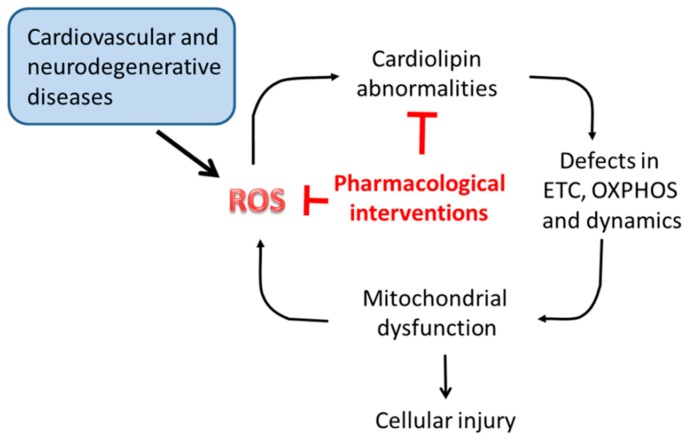
Cardiolipin as target for pharmacological intervention in disease. ETC, electron transport chain; OXPHOS, oxidative phosphorylation; ROS, reactive oxygen species. For details, see the text.

Mitochondrial dysfunction is considered the primary cause of secondary complications in several diseases, both rare and common. Due to the central role of CL in multiple biochemical reactions and processes of mitochondrial metabolism and dynamics, alterations occurring in its content, fatty acyl chains composition and level of oxidation may dramatically affect mitochondrial function, and hence cell viability. As discussed in the previous sections, CL alterations underlay mitochondrial dysfunction associated with several pathophysiological situations and disorders, including Barth syndrome, myocardial ischemia/reperfusion, metabolic disorders and neurodegenerative diseases. Thus, compounds able to preserve mitochondrial CL content and integrity would be useful in preventing or attenuate mitochondrial damages associated with these diseases (Figure 4). Several agents targeting directly or indirectly mitochondrial CL, have been shown to be effective in preventing mitochondrial dysfunction and dynamics, thus potentially beneficial for these diseases. Some of these compounds are currently being tested in clinical trials.

Elamipretide (previously referred to as Bendavia, SS-31, MPT-131) is a water-soluble tetrapeptide that can directly target mitochondrial membranes [129,130]. This peptide is able to access the IMM and selectively bind to CL through electrostatic and hydrophobic interactions. The binding of elamipretide to CL prevents the formation of the complex cyt c-CL and its associated peroxidase activity, thus reducing ROS-mediated CL peroxidation, while improving cyt c electron transfer function [129,130]. The interaction of elamipretide with CL is supposed to preserve the integrity and proper morphology of mitochondrial cristae and to stabilize the ETC supercomplexes, thus increasing respiratory function and energy production by the OXPHOS process, while minimizing ROS production [129,130]. Elamipretide appears to be also involved in modulation of mitochondrial quality control and mitophagy [131]. It was observed that this peptide selectively stimulates the engulfment of mitochondria into autophagosomes and prevents its defects induced by nutrient excess in INS1 β-cells [131]. Several studies have demonstrated that elamipretide consistently improves mitochondrial, cellular and organ function in both in vitro and in vivo disease models for which mitochondrial dysfunction and CL alterations are understood to be an important causative component [33,129,130]. In animal experimental studies, elamipretide was shown to provide myocardial protection against I/R injury [132]. In fact, when administered at the onset of reperfusion, this peptide reduced myocardial infarct (MI) and prevented adverse left ventricular remodeling after MI. Furthermore, elamipretide improved post-infarction myocardial function, restored mitochondrial energy metabolism gene expression and suppressed cardiac fibrosis in the border zone of the infarcted heart [132]. Abnormalities in cardiolipin composition are implicated in mitochondrial dysfunction and consequent impaired energy production occurring in heart failure, leading to heightened interest in mitochondrial function as therapeutic target [133]. Very recently, the effects of elamipretide on mitochondrial function in failing human hearts ex vivo was investigated [134]. Using freshly explanted human hearts, it was shown that elamipretide significantly improved impaired mitochondrial function in HF, enhancing mitochondrial oxygen flux, complex I and IV activities and supercomplex-associated CIV activity [134]. In addition, elamipretide was found to increase exercise performance after 5 days of treatment in patients with primary mitochondrial myopathy [135]. These findings remain to be tested in larger trials with longer treatment periods to detect other potential therapeutic benefits in individuals affected by these pathological conditions.

Melatonin, the major secretory product of the pineal gland, involved in the regulation of sleep and modulation of circadian rhythms, is also recognized as a powerful antioxidant agent [136]. The free radical scavenging properties of this molecule can be mainly ascribed to its electron-rich aromatic indole ring, which makes it a potent electron donor, thus lowering oxidative stress. Melatonin is synthesized and highly concentrated inside mitochondria, the major source of free radicals, where it exerts an effective antioxidant activity, thus preserving and improving mitochondrial bioenergetic function [137]. Furthermore, this indoleamine has been validated to maintain a healthy mitochondrial network by modulating mitochondrial dynamics, biogenesis and mitophagy [136]. As reported above, ROS-induced CL oxidation affects several parameters of mitochondrial bioenergetics, including mPTP opening and cyt c release from mitochondria, two important events involved in the apoptotic process. Our in vitro studies on isolated rat heat heart mitochondria, have shown that melatonin inhibits both mPTP opening and cyt c release from mitochondria [138]. These protective effects of melatonin may be reasonably explained by the ability of this indolamine to prevent CL oxidation and, more specifically, the peroxidation of linoleic fatty acyl chains, which are the main constituents of heart CL molecules. This is also supported by the finding that melatonin displays strong in vitro lipid peroxyl radicals (LOO^.^) scavenging properties [139]. Consistent with these results, melatonin treatment was shown to have strong protective effect against mitochondrial dysfunction in a rat model of heart I/R, lowering ROS production, preventing alterations in both ETC respiratory complexes function and cardiolipin profile and inhibiting mPTP opening and cyt c release [138]. These effects may account for the improvement of post-ischemic hemodynamic function and for the reduction in the infarct size and necrotic damage, observed in melatonin-treated rat heart upon reperfusion, [138]. These results were translated in a clinical setting. It was shown that melatonin administration to patients with ST-segment elevation myocardial infarction, resulted in a significant reduction in the infarct size after primary percutaneous coronary intervention [140]. Thus, melatonin treatment may represent an effective therapeutic strategy to combat pathophysiological situations and diseases in which oxidative stress, mitochondrial dysfunction and cardiolipin abnormalities are understood to play a causative role, including, in addition to myocardial I/R, neurodegenerative disorders, such as Parkinson’s and Alzheimer diseases [141].

Cationic derivatives of plastoquinones are a class of amphiphilic synthetic compounds with strong antioxidant properties [142]. Due to their biochemical and biophysical characteristics, these compounds can easily penetrate the phospholipid bilayer of mitochondrial membranes where they are selectively accumulated. Inside mitochondria these compounds exert their powerful antioxidant activity, preventing or lowering mitochondrial oxidative damages. One of the mechanisms proposed for the protective action of plastoquinone derivatives against mitochondrial-induced oxidative damages, is their ability to preserve CL integrity, by preventing its oxidation by ROS attack [143]. CL oxidation is implicated in mitochondrial dysfunction and apoptosis; thus, the ability of plastoquinones to inhibit CL oxidation may result in a protection of mitochondrial function and inhibition of the apoptotic process. These protective effects of plastoquinones may explain their beneficial effects against several pathophysiological situations and diseases [144].

## 10. Conclusions and Perspectives

It is now widely accepted that cardiolipin plays a central role in mitochondrial metabolism, by maintaining the proper architecture and morphology of the mitochondrial membranes and by regulating the activity of a variety of proteins and enzymes involved in mitochondrial function. CL interacts with and is required for full activity of respiratory chain complexes, as well as for their assembly and stabilization into supercomplexes, essential for a more efficient electron/proton flux, and hence for ATP synthesis. How CL modulates the activity of these proteins and which role this phospholipid plays in the supercomplexes assembly and stabilization, are questions which still await a satisfying explanation, and this remains an exciting area for future investigation. CL is also emerging as an important player in mitochondrial fusion-division dynamics, in mitochondrial biogenesis and proteins import and in regulation of mitophagy and cell death processes. Several factors are involved in mitochondrial dynamics, cristae formation and CL biosynthesis and remodeling; however, it is still unknown how these events are interconnected and cooperate. Due to their high content of unsaturated acyl chains, CL molecules are readily susceptible to ROS attack. The oxidation of CL, by altering its biophysical properties, perturbs the interaction of this phospholipid with host proteins, impairing their stability and function. CL oxidation and abnormalities occurring in its content and acyl chain composition, due to defects in biosynthesis and remodeling, may result in aberrant mitochondrial function and dynamics with important implications in several pathophysiological situations and diseases, including Barth syndrome, myocardial I/R injury, heart failure, diabetes and neurodegenerative diseases. Compounds able to prevent CL abnormalities and hence, dysregulation in mitochondrial function and dynamics, would be beneficial in managing these diseases. Several agents directly or indirectly targeting CL, including elamipretide, melatonin and plastoquinone derivatives, have been proven to be effective in preserving CL integrity, thus protecting mitochondrial function and dynamics and opening new perspectives for treatment of these disorders. Our knowledge of how CL abnormalities contribute to disease pathogenesis and progression remains rudimentary. The recent establishment of new model systems, including drosophila, zebrafish, mouse models, cultured mammalian cells and yeast [145], will contribute to answer to this and additional questions and will highlight the role of cardiolipin in mitochondrial function and dynamics in health and disease.

## References

[1] Osellame L.D., Blacker T.S., Duchen M.R. (2012). Cellular and molecular mechanisms of mitochondrial function. Best Pr. Res. Clin. Endocrinol. Metab..

[2] Horvath S.E., Daum G. (2013). Lipids of mitochondria. Prog. Lipid Res..

[3] Mejia E.M., Hatch G.M. (2016). Mitocho, ndrial phospholipids: role in mitochondrial function. J. Bioenerg. Biomembr..

[4] Lu Y.W., Claypool S.M. (2015). Disorders of phospholipid metabolism: an emerging class of mitochondrial disease due to defects in nuclear genes. Front. Genet..

[5] Ren M., Phoon C.K., Schlame M. (2014). Metabolism and function of mitochondrial cardiolipin. Prog. Lipid Res..

[6] Ball W.B., Neff J.K., Gohil V.M. (2017). The role of nonbilayer phospholipids in mitochondrial structure and function. FEBS Lett..

[7] Elías-Wolff F., Lindén M., Lyubartsev A.P., Brandt E.G. (2019). Curvature sensing by cardiolipin in simulated buckled membranes. Soft Matter.

[8] Claypool S.M., Koehler C.M. (2012). The complexity of cardiolipin in health and disease. Trends Biochem. Sci..

[9] Paradies G., Paradies V., De Benedictis V., Ruggiero F.M., Petrosillo G. (2014). Functional role of cardiolipin in mitochondrial bioenergetics. Biochim. Biophys. Acta (BBA)-Bioenerg..

[10] Dudek J. (2017). Role of Cardiolipin in Mitochondrial Signaling Pathways. Front. Cell Dev. Boil..

[11] Musatov A., Sedlák E. (2017). Role of cardiolipin in stability of integral membrane proteins. Biochimie.

[12] Ikon N., Ryan R.O. (2017). Cardiolipin and mitochondrial cristae organization. Biochim. Biophys. Acta (BBA)-Biomembr..

[13] Kameoka S., Adachi Y., Okamoto K., Iijima M., Sesaki H. (2018). Phosphatidic Acid and Cardiolipin Coordinate Mitochondrial Dynamics. Trends Cell Biol..

[14] Ban T., Ishihara T., Kohno H., Saita S., Ichimura A., Maenaka K., Oka T., Mihara K., Ishihara N. (2017). Molecular basis of selective mitochondrial fusion by heterotypic action between OPA1 and cardiolipin. Nature.

[15] Malhotra K., Modak A., Nangia S., Daman T.H., Gunsel U., Robinson V.L., Mokranjac D., May E.R., Alder N.N. (2017). Cardiolipin mediates membrane and channel interactions of the mitochondrial TIM23 protein import complex receptor Tim50. Sci. Adv..

[16] Gohil V.M., Greenberg M.L. (2009). Mitochondrial membrane biogenesis: phospholipids and proteins go hand in hand: Figure 1. J. Cell Boil..

[17] Schlattner U., Tokarska-Schlattner M., Epand R.M., Boissan M., Lacombe M.L., Kagan V.E. (2018). NME4/nucleoside diphosphate kinase D in cardiolipin signaling and mitophagy. Lab. Invest..

[18] Dudek J., Hartmann M., Rehling P. (2019). The role of mitochondrial cardiolipin in heart function and its implication in cardiac disease. Biochim. et Biophys. Acta (BBA) - Mol. Basis Dis..

[19] Ott M., Zhivotovsky B., Orrenius S. (2007). Role of cardiolipin in cytochrome c release from mitochondria. Cell Death Differ..

[20] Schug Z.T., Gottlieb E. (2009). Cardiolipin acts as a mitochondrial signaling platform to launch apoptosis. Biochim. Biophys. Acta. (BBA)-Biomembr..

[21] Lucken-Ardjomande S., Montessuit S., Martinou J.C. (2008). Contributions to Bax insertion and oligomerization of lipids of the mitochondrial outer membrane. Cell Death Differ..

[22] Paradies G., Petrosillo G., Paradies V., Ruggiero F.M. (2009). Role of cardiolipin peroxidation and Ca^2+^ in mitochondrial dysfunction and disease. Cell calcium.

[23] Paradies G., Paradies V., Ruggiero F.M., Petrosillo G. (2014). Cardiolipin and mitochondrial function in health and disease. Antioxid. Redox Signal..

[24] Kagan V.E., Chu C.T., Tyurina Y.Y., Cheikhi A., Bayir H. (2014). Cardiolipin asymmetry, oxidation and signaling. Chem. Phys. Lipids.

[25] Jalmar O., François-Moutal L., García-Sáez A.J., Perry M., Granjon T., Gonzalvez F., Gottlieb E., Ayala-Sanmartin J., Klösgen B., Schwille P. (2013). Caspase-8 binding to cardiolipin in giant unilamellar vesicles provides a functional docking platform for bid. PLoS ONE.

[26] Petrosillo G., Casanova G., Matera M., Ruggiero F.M., Paradies G. (2006). Interaction of peroxidized cardiolipin with rat-heart mitochondrial membranes: induction of permeability transition and cytochrome c release. FEBS Lett..

[27] Chicco A.J., Sparagna G.C. (2007). Role of cardiolipin alterations in mitochondrial dysfunction and disease. Am. J. Physiol. Cell Physiol..

[28] Han X., Yang J., Yang K., Zhongdan Z., Abendschein D.R., Gross R.W. (2007). Alterations in Myocardial Cardiolipin Content and Composition Occur at the Very Earliest Stages of Diabetes: A Shotgun Lipidomics Study. Biochemistry.

[29] Gaspard G.J., McMaster C.R. (2015). Cardiolipin metabolism and its causal role in the etiology of the inherited cardiomyopathy Barth syndrome. Chem. Phys. Lipids.

[30] Lesnefsky E.J., Chen Q., Tandler B., Hoppel C.L. (2017). Mitochondrial Dysfunction and Myocardial Ischemia-Reperfusion: Implications for Novel Therapies. Annu. Rev. Pharmacol. Toxicol..

[31] Pointer C.B., Klegeris A. (2017). Cardiolipin in Central Nervous System Physiology and Pathology. Cell Mol. Neurobiol..

[32] Paradies G., Paradies V., Ruggiero F.M., Petrosillo G. (2018). Mitochondrial bioenergetics and cardiolipin alterations in myocardial ischemia-reperfusion injury: implications for pharmacological cardioprotection. Am. J. Physiol. Circ. Physiol..

[33] Pennington E.R., Funai K., Brown D.A., Shaikh S.R. (2019). The role of cardiolipin concentration and acyl chain composition on mitochondrial inner membrane molecular organization and function. Biochim. et Biophys. Acta (BBA) - Mol. Cell Boil. Lipids.

[34] Mejia E.M., Nguyen H., Hatch G.M. (2014). Mammalian cardiolipin biosynthesis. Chem. Phys. Lipids.

[35] Schlame M., Ren M. (2006). Barth syndrome, a human disorder of cardiolipin metabolism. FEBS Lett..

[36] Clarke S.L., Bowron A., Gonzalez I.L., Groves S.J., Newbury-Ecob R., Clayton N., Martin R.P., Tsai-Goodman B., Garratt V., Ashworth M. (2013). Barth syndrome. Orphanet J. Rare Dis..

[37] Ikon N., Ryan R.O. (2017). Barth Syndrome: Connecting Cardiolipin to Cardiomyopathy. Lipids.

[38] Schlattner U., Tokarska-Schlattner M., Rousseau D., Boissan M., Mannella C., Epand R., Lacombe M.-L. (2014). Mitochondrial cardiolipin/phospholipid trafficking: The role of membrane contact site complexes and lipid transfer proteins. Chem. Phys. Lipids.

[39] Liu J., Dai Q., Chen J., Durrant D., Freeman A., Liu T., Grossman D., Lee R.M. (2003). Phospholipid scramblase 3 controls mitochondrial structure, function, and apoptotic response. Mol. Cancer Res..

[40] Epand R.F., Tokarska-Schlattner M., Schlattner U., Wallimann T., Epand R.M. (2007). Cardiolipin clusters and membrane domain formation induced by mitochondrial proteins. J. Mol. Biol..

[41] Palmieri F., Pierri C.L. (2010). Mitochondrial metabolite transport. Essays Biochem..

[42] Palmieri F., Monné M. (2016). Discoveries, metabolic roles and diseases of mitochondrial carriers: A review. Biochim. et Biophys. Acta (BBA) - Bioenerg..

[43] Klingenberg M. (2009). Cardiolipin and mitochondrial carriers. Biochim. Biophys. Acta..

[44] Nury H., Dahout-Gonzalez C., Trezeguet V., Lauquin G., Brandolin G., Pebay-Peyroula E. (2006). Relations Between Structure and Function of the Mitochondrial ADP/ATP Carrier. Annu. Rev. Biochem..

[45] Lange C., Nett J.H., Trumpower B.L., Hunte C. (2001). Specific roles of protein–phospholipid interactions in the yeast cytochrome bc1 complex structure. EMBO J..

[46] Musatov A., Robinson N.C. (2014). Bound Cardiolipin Is Essential for Cytochrome c Oxidase Proton Translocation. Biochemistry.

[47] Pöyry S., Cramariuc O., Postila P.A., Kaszuba K., Sarewicz M., Osyczka A., Vattulainen I., Rog T. (2013). Atomistic simulations indicate cardiolipin to have an integral role in the structure of the cytochrome bc1 complex. Biochim. et Biophys. Acta (BBA) - Gen. Subj..

[48] Enríquez J.A. (2016). Supramolecular Organization of Respiratory Complexes. Annu. Rev. Physiol..

[49] Genova M.L., Lenaz G. (2014). Functional role of mitochondrial respiratory supercomplexes. Biochim. Biophys. Acta (BBA)-Bioenerg..

[50] A Letts J., A Sazanov L. (2017). Clarifying the supercomplex: the higher-order organization of the mitochondrial electron transport chain. Nat. Struct. Mol. Boil..

[51] Schägger H., Pfeiffer K. (2000). Supercomplexes in the respiratory chains of yeast and mammalian mitochondria. EMBO J..

[52] Claypool S.M. (2009). Cardiolipin, a critical determinant of mitochondrial carrier protein assembly and function. Biochim. et Biophys. Acta (BBA) - Biomembr..

[53] Mileykovskaya E., Dowhan W. (2014). Cardiolipin-dependent formation of mitochondrial respiratory supercomplexes. Chem. Phys. Lipids.

[54] Zhou D.R., Eid R., Boucher E., Miller K.A., Mandato C.A., Greenwood M.T. (2019). Stress is an agonist for the induction of programmed cell death: A review. Biochim. et Biophys. Acta (BBA) - Bioenerg..

[55] Murphy M.P. (2009). How mitochondria produce reactive oxygen species. Biochem. J..

[56] Paradies G., Petrosillo G., Pistolese M., Ruggiero F.M. (2000). The effect of reactive oxygen species generated from the mitochondrial electron transport chain on the cytochrome c oxidase activity and on the cardiolipin content in bovine heart submitochondrial particles. FEBS Lett..

[57] Paradies G., Petrosillo G., Pistolese M., Ruggiero F.M. (2001). Reactive oxygen species generated by the mitochondrial respiratory chain affect the complex III activity via cardiolipin peroxidation in beef-heart submitochondrial particles. Mitochondrion.

[58] Paradies G., Petrosillo G., Pistolese M., Ruggiero F.M. (2002). Reactive oxygen species affect mitochondrial electron transport complex I activity through oxidative cardiolipin damage. Gene.

[59] Xiao M., Zhong H., Xia L., Tao Y., Yin H. (2017). Pathophysiology of mitochondrial lipid oxidation: Role of 4-hydroxynonenal (4-HNE) and other bioactive lipids in mitochondria. Free Radic. Biol. Med..

[60] Uchida K. (2003). 4-Hydroxy-2-nonenal: a product and mediator of oxidative stress. Prog. Lipid Res..

[61] Quintana-Cabrera R., Mehrotra A., Rigoni G., Soriano M. (2018). Who and how in the regulation of mitochondrial cristae shape and function. Biochem. Biophys. Res. Commun..

[62] Rampelt H., Zerbes R.M., van der Laan M., Pfanner N. (2017). Role of the mitochondrial contact site and cristae organizing system in membrane architecture and dynamics. Biochim. Biophys. Acta (BBA)-Mol. Cell Res..

[63] Haines T.H., A Dencher N. (2002). Cardiolipin: a proton trap for oxidative phosphorylation. FEBS Lett..

[64] Acehan D., Malhotra A., Xu Y., Ren M., Stokes D.L., Schlame M. (2011). Cardiolipin Affects the Supramolecular Organization of ATP Synthase in Mitochondria. Biophys. J..

[65] Halestrap A.P. (2009). What is the mitochondrial permeability transition pore?. J. Mol. Cell. Cardiol..

[66] Bernardi P., Di Lisa F. (2015). The mitochondrial permeability transition pore: Molecular nature and role as a target in cardioprotection. J. Mol. Cell. Cardiol..

[67] Biasutto L., Azzolini M., Szabò I., Zoratti M. (2016). The mitochondrial permeabilità transition pore in AD 2016: An update. Biochim. Biophys. Acta (BBA)-Mol. Cell Res..

[68] Carraro M., Checchetto V., Szabò I., Bernardi P. (2019). F-ATP Synthase and the Permeability Transition Pore: Fewer doubts, more certainties. FEBS Lett..

[69] Eble K.S., Coleman W.B., Hantgan R.R., Cunningham C.C. (1990). Tightly associated cardiolipin in the bovine heart mitochondrial ATP synthase as analyzed by 31P nuclear magnetic resonance spectroscopy. J. Boil. Chem..

[70] Duncan A.L., Robinson A.J., Walker J.E. (2016). Cardiolipin binds selectively but transiently to conserved lysine residues in the rotor of metazoan ATP synthases. Proc. Natl. Acad. Sci..

[71] Giorgio V., von Stockum S., Antoniel M., Fabbro A., Fogolari F., Forte M., Glick G.D., Petronilli V., Zoratti M., Szabó I. (2013). Dimers of mitochondrial ATP synthase form the permeability transition pore. Proc. Natl. Acad. Sci..

[72] Halestrap A.P., Richardson A.P. (2015). The mitochondrial permeability transition: A current perspective on its identity and role in ischaemia/reperfusion injury. J. Mol. Cell. Cardiol..

[73] Karch J., Molkentin J.D. (2014). Identifying the components of the elusive mitochondrial permeability transition pore. Proc. Natl. Acad. Sci..

[74] Claypool S.M., Oktay Y., Boontheung P., Loo J.A., Koehler C.M. (2008). Cardiolipin defines the interactome of the major ADP/ATP carrier protein of the mitochondrial inner membrane. J. Cell Biol..

[75] Halestrap A.P. (2014). The C Ring of the F1Fo ATP Synthase Forms the Mitochondrial Permeability Transition Pore: A Critical Appraisal. Front. Oncol..

[76] Hüttemann M., Pecina P., Rainbolt M., Sanderson T.H., Kagan V.E., Samavati L., Doan J.W., Lee I. (2011). The multiple functions of cytochrome c and their regulation in life and death decisions of the mammalian cell: From respiration to apoptosis. Mitochondrion.

[77] Pereverzev M.O., Vygodina T.V., Konstantinov A.A., Skulachev V.P. (2003). Cytochrome c, an ideal antioxidant. Biochem. Soc. Trans..

[78] Tuominen E.K.J., Wallace C.J.A., Kinnunen P.K.J. (2002). Phospholipid-Cytochrome c Interaction: EVIDENCE FOR THE EXTENDED LIPID ANCHORAGE. J. Boil. Chem..

[79] Sinibaldi F., Howes B.D., Piro M.C., Polticelli F., Bombelli C., Ferri T., Coletta M., Smulevich G., Santucci R. (2010). Extended cardiolipin anchorage to cytochrome c: a model for protein–mitochondrial membrane binding. JBIC J. Boil. Inorg. Chem..

[80] Kagan V.E., Tyurin V.A., Jiang J., Tyurina Y.Y., Ritov V.B., Amoscato A.A., Osipov A.N., Belikova N.A., Kapralov A.A., Kini V. (2005). Cytochrome c acts as a cardiolipin oxygenase required for release of proapoptotic factors. Nat. Chem. Biol..

[81] Belikova N.A., Vladimirov Y.A., Osipov A.N., Kapralov A.A., Tyurin V.A., Potapovich M.V., Basova L.V., Peterson J., Kurnikov I.V., Kagan V.E. (2006). Peroxidase Activity and Structural Transitions of Cytochrome c Bound to Cardiolipin-Containing Membranes. Biochemistry.

[82] Petrosillo G., Ruggiero F.M., Pistolese M., Paradies G. (2001). Reactive oxygen species generated from the mitochondrial electron transport chain induce cytochrome c dissociation from beef-heart submitochondrial particles via cardiolipin peroxidation. Possible role in the apoptosis. FEBS Lett..

[83] Gonzalvez F., Schug Z.T., Houtkooper R.H., MacKenzie E.D., Brooks D.G., Wanders R.J., Petit P.X., Vaz F.M., Gottlieb E. (2008). Cardiolipin provides an essential activating platform for caspase-8 on mitochondria. J. Cell Biol..

[84] Kiriyama Y., Nochi H. (2017). Intra- and Intercellular Quality Control Mechanisms of Mitochondria. Cells.

[85] Tilokani L., Nagashima S., Paupe V., Prudent J. (2018). Mitochondrial dynamics: overview of molecular mechanisms. Essays Biochem..

[86] Kraus F., Ryan M.T. (2017). The constriction and scission machineries involved in mitochondrial fission. J. Cell Sci..

[87] Kojima R., Kakimoto Y., Furuta S., Itoh K., Sesaki H., Endo T., Tamura Y. (2019). Maintenance of Cardiolipin and Crista Structure Requires Cooperative Functions of Mitochondrial Dynamics and Phospholipid Transport. Cell Rep..

[88] Li X.-X., Tsoi B., Li Y.-F., Kurihara H., He R.-R. (2015). Cardiolipin and Its Different Properties in Mitophagy and Apoptosis. J. Histochem. Cytochem..

[89] Maguire J.J., Tyurina Y.Y., Mohammadyani D., Kapralov A.A., Anthonymuthu T.S., Qu F., Amoscato A.A., Sparvero L.J., Tyurin V.A., Planas-Iglesias J. (2017). Known unknowns of cardiolipin signaling: The best is yet to come. Biochim. Biophys. Acta (BBA)-Mol. Cell Biol. Lipids.

[90] Stepanyants N., Macdonald P.J., Francy C.A., Mears J.A., Qi X., Ramachandran R. (2015). Cardiolipin’s propensity for phase transition and its reorganization by dynamin-related protein 1 form a basis for mitochondrial membrane fission. Mol. Biol. Cell.

[91] Bustillo-Zabalbeitia I., Montessuit S., Raemy E., Basáñez G., Terrones O., Martinou J.-C. (2014). Specific Interaction with Cardiolipin Triggers Functional Activation of Dynamin-Related Protein 1. PLOS ONE.

[92] Lemasters J.J. (2005). Selective Mitochondrial Autophagy, or Mitophagy, as a Targeted Defense Against Oxidative Stress, Mitochondrial Dysfunction, and Aging. Rejuvenation Res..

[93] Youle R.J., Van Der Bliek A.M. (2012). Mitochondrial Fission, Fusion, and Stress. Science.

[94] Chu C.T., Ji J., Dagda R.K., Jiang J.F., Tyurina Y.Y., Kapralov A.A., Tyurin V.A., Yanamala N., Shrivastava I.H., Mohammadyani D. (2013). Cardiolipin externalization to the outer mitochondrial membrane acts as an elimination signal for mitophagy in neuronal cells. Nature.

[95] Huang W., Choi W., Hu W., Mi N., Guo Q., Ma M., Liu M., Tian Y., Lu P., Wang F.-L. (2012). Crystal structure and biochemical analyses reveal Beclin 1 as a novel membrane binding protein. Cell Res..

[96] Ades L.C., Gedeon A.K., Wilson M.J., Latham M., Partington M.W., Mulley J.C., Nelson J., Lui K., Sillence D.O. (1993). Barth syndrome: Clinical features and confirmation of gene localisation to distal Xq28. Am. J. Med Genet..

[97] Bione S., D’Adamo P., Maestrini E., Gedeon A.K., Bolhuis P.A., Toniolo D. (1996). A novel X-linked gene, G4.5. is responsible for Barth syndrome. Nat. Genet..

[98] McKenzie M., Lazarou M., Thorburn D.R., Ryan M.T. (2006). Mitochondrial Respiratory Chain Supercomplexes Are Destabilized in Barth Syndrome Patients. J. Mol. Boil..

[99] Mejia E.M., Cole L.K., Hatch G.M. (2014). Cardiolipin metabolism and the role it plays in heart failure and mitochondrial supercomplex formation. Cardiovasc. Hematol. Disord.-Drug Targets.

[100] Huang Y., Powers C., Madala S.K., Greis K.D., Haffey W.D., Towbin J.A., Purevjav E., Javadov S., Strauss A.W., Khuchua Z. (2015). Cardiac metabolic pathways affected in the mouse model of barth syndrome. PLoS One.

[101] Acehan D., Xu Y., Stokes D.L., Schlame M. (2007). Comparison of lymphoblast mitochondria from normal subjects and patients with Barth syndrome using electron microscopic tomography. Lab. Invest..

[102] Yellon D.M., Hausenloy D.J. (2007). Myocardial reperfusion injury. New Engl. J. Med..

[103] Murphy E., Steenbergen C. (2008). Mechanisms underlying acute protection from cardiac ischemia-reperfusion injury. Physiol. Rev..

[104] Consolini A.E., Ragone M.I., Bonazzola P., Colareda G.A. (2017). Mitochondrial Bioenergetics During Ischemia and Reperfusion. Mitochondrial Dyn. Cardiovasc. Med..

[105] Chen Y.R., Zweier J.L. (2014). Cardiac mitochondria and reactive oxygen species generation. Circ. Res..

[106] Granger D.N., Kvietys P.R. (2015). Reperfusion injury and reactive oxygen species: The evolution of a concept. Redox Boil..

[107] Zhou T., Chuang C.C., Zuo L. (2015). Molecular Characterization of Reactive Oxygen Species in Myocardial Ischemia-Reperfusion Injury. Biomed Res. Int..

[108] Paradies G., Petrosillo G., Pistolese M., Di Venosa N., Serena D., Ruggiero F.M. (1999). Lipid peroxidation and alterations to oxidative metabolism in mitochondria isolated from rat heart subjected to ischemia and reperfusion. Free Radic. Biol. Med..

[109] Maneechote C., Palee S., Chattipakorn S.C., Chattipakorn N. (2017). Roles of mitochondrial dynamics modulators in cardiac ischaemia/reperfusion injury. J. Cell Mol. Med..

[110] Ma Z.A., Zhao Z., Turk J. (2012). Mitochondrial dysfunction and β-cell failure in type 2 diabetes mellitus. Exp. Diabetes. Res..

[111] Bugger H., Abel E.D. (2010). Mitochondria in the diabetic heart. Cardiovasc. Res..

[112] Diaz-Morales N., Rovira-Llopis S., Escribano-Lopez I., Bañuls C., Lopez-Domenech S., Falcón R., De Maranon A.M., Sola E., Jover A., Roldan I. (2016). Role of Oxidative Stress and Mitochondrial Dysfunction in Skeletal Muscle in Type 2 Diabetic Patients. Curr. Pharm. Des..

[113] Shi Y. (2010). Emerging roles of cardiolipin remodeling in mitochondrial dysfunction associated with diabetes, obesity, and cardiovascular diseases. J. Biomed. Res..

[114] Chang W., Hatch G.M., Wang Y., Yu F., Wang M. (2019). The relationship between phospholipids and insulin resistance: From clinical to experimental studies. J. Cell Mol. Med..

[115] Jheng H.F., Tsai P.J., Guo S.M., Kuo L.H., Chang C.S., Su I.J., Chang C.R., Tsai Y.S. (2012). Mitochondrial fission contributes to mitochondrial dysfunction and insulin resistance in skeletal muscle. Mol. Cell Biol..

[116] Filippi B.M., Abraham M.A., Silva P.N., Rasti M., Lapierre M.P., Bauer P.V., Rocheleau J.V., Lam T.K. (2017). Dynamin-Related Protein 1-Dependent Mitochondrial Fission Changes in the Dorsal Vagal Complex Regulate Insulin Action. Cell Rep..

[117] Kalia L.V., Lang A.E. (2015). Parkinson’s disease. Lancet.

[118] Dexter D.T., Jenner P. (2013). Parkinson disease: from pathology to molecular disease mechanisms. Free. Radic. Boil. Med..

[119] Xicoy H., Wieringa B., Martens G.J.M. (2019). The Role of Lipids in Parkinson’s Disease. Cells.

[120] Ghio S., Kamp F., Cauchi R., Giese A., Vassallo N. (2016). Interaction of α-synuclein with biomembranes in Parkinson’s disease —role of cardiolipin. Prog. Lipid Res..

[121] Ellis C.E., Murphy E.J., Mitchell D.C., Golovko M.Y., Scaglia F., Barceló-Coblijn G.C., Nussbaum R.L. (2005). Mitochondrial lipid abnormality and electron transport chain impairment in mice lacking alpha-synuclein. Mol. Cell Biol..

[122] Lopez-Fabuel I., Martin-Martin L., Resch-Beusher M., Azkona G., Sanchez-Pernaute R., Bolaños J.P. (2017). Mitochondrial respiratory chain disorganization in Parkinson’s disease-relevant PINK1 and DJ1 mutants. Neurochem. Int..

[123] Bayir H., Kapralov A.A., Jiang J., Huang Z., Tyurina Y.Y., Tyurin V.A., Zhao Q., Belikova N.A., Vlasova I.I., Maeda A. (2009). Peroxidase mechanism of lipid-dependent cross-linking of synuclein with cytochrome C: protection against apoptosis versus delayed oxidative stress in Parkinson disease. J. Biol. Chem..

[124] Perier C., Tieu K., Guegan C., Caspersen C., Jackson-Lewis V., Carelli V., Vila M. (2005). Complex I deficiency primes Bax-dependent neuronal apoptosis through mitochondrial oxidative damage. Proc. Natl. Acad. Sci..

[125] Petrosillo G., Matera M., Casanova G., Ruggiero F., Paradies G. (2008). Mitochondrial dysfunction in rat brain with agingInvolvement of complex I, reactive oxygen species and cardiolipin. Neurochem. Int..

[126] Pickrell A.M., Youle R.J. (2015). The Roles of PINK1, Parkin and Mitochondrial Fidelity in Parkinson’s Disease. Neuron.

[127] Chu C.T., Bayir H., Kagan V.E. (2014). LC3 binds externalized cardiolipin on injured mitochondria to signal mitophagy in neurons: Implications for Parkinson disease. Autophagy.

[128] Song C., Zhang J., Qi S., Liu Z., Zhang X., Zheng Y., Andersen J.-P., Zhang W., Strong R., Martinez P.A. (2019). Cardiolipin remodeling by ALCAT1 links mitochondrial dysfunction to Parkinson’s diseases. Aging Cell.

[129] Szeto H.H. (2014). First-in-class cardiolipin-protective compound as a therapeutic agent to restore mitochondrial bioenergetics. Br. J. Pharmacol..

[130] Szeto H.H., Birk A.V. (2014). Serendipity and the Discovery of Novel Compounds That Restore Mitochondrial Plasticity. Clin. Pharmacol. Ther..

[131] Petcherski A., Trudeau K.M., Wolf D.M., Segawa M., Lee J., Taddeo E.P., Deeney J.T., Liesa M. (2018). Elamipretide Promotes Mitophagosome Formation and Prevents Its Reduction Induced by Nutrient Excess in INS1 β-cells. J. Mol. Boil..

[132] Dai W., Shi J., Gupta R.C., Sabbah H.N., Hale S.L., Kloner R.A. (2014). Bendavia, a Mitochondria-targeting Peptide, Improves Postinfarction Cardiac Function, Prevents Adverse Left Ventricular Remodeling, and Restores Mitochondria-related Gene Expression in Rats. J. Cardiovasc. Pharmacol..

[133] Dolinsky V.W., Cole L.K., Sparagna G.C., Hatch G.M. (2016). Cardiac mitochondrial energy metabolism in heart failure: Role of cardiolipin and sirtuins. Biochim. et Biophys. Acta (BBA) - Mol. Cell Boil. Lipids.

[134] Chatfield K.C., Sparagna G.C., Chau S., Phillips E.K., Ambardekar A.V., Aftab M., Mitchell M.B., Sucharov C.C., Miyamoto S.D., Stauffer B.L. (2019). Elamipretide Improves Mitochondrial Function in the Failing Human Heart. JACC: Basic Transl. Sci..

[135] Karaa A., Haas R., Goldstein A., Vockley J., Weaver W.D., Cohen B.H. (2018). Randomized dose-escalation trial of elamipretide in adults with primary mitochondrial myopathy. Neurology.

[136] Zhao D., Yu Y., Shen Y., Liu Q., Zhao Z., Sharma R., Reiter R.J. (2019). Melatonin Synthesis and Function: Evolutionary History in Animals and Plants. Front. Endocrinol..

[137] Tan D.-X., Manchester L.C., Qin L., Reiter R.J. (2016). Melatonin: A Mitochondrial Targeting Molecule Involving Mitochondrial Protection and Dynamics. Int. J. Mol. Sci..

[138] Petrosillo G., Moro N., Ruggiero F.M., Paradies G. (2009). Melatonin inhibits cardiolipin peroxidation in mitochondria and prevents the mitochondrial permeability transition and cytochrome c release. Free Radic. Biol. Med..

[139] Mekhloufi J., Bonnefont-Rousselot D., Yous S., Lesieur D., Couturier M., Thérond P., Legrand A., Jore D., Gardès-Albert M. (2005). Antioxidant activity of melatonin and a pinoline derivative on linoleate model system. J. Pineal Res..

[140] Dominguez-Rodriguez A., Abreu-Gonzalez P., de la Torre-Hernandez J.M., Consuegra-Sanchez L., Piccolo R., Gonzalez-Gonzalez J., Garcia-Camarero T., Del Mar Garcia-Saiz M., Aldea-Perona A., Reiter R.J. (2017). Usefulness of Early Treatment With Melatonin to Reduce Infarct Size in Patients With ST-Segment Elevation Myocardial Infarction Receiving Percutaneous Coronary Intervention (From the Melatonin Adjunct in the Acute Myocardial Infarction Treated With Angioplasty Trial). Am. J. Cardiol..

[141] Wongprayoon P., Govitrapong P. (2017). Melatonin as a mitochondrial protector in neurodegenerative diseases. Cell. Mol. Life Sci..

[142] Feniouk B.A., Skulachev V.P. (2017). Cellular and Molecular Mechanisms of Action of Mitochondria-Targeted Antioxidants. Curr. Aging Sci..

[143] Skulachev V.P., Antonenko Y.N., Cherepanov D.A., Chernyak B.V., Izyumov D.S., Khailova L.S., Klishin S.S., Korshunova G.A., Lyamzaev K.G., Pletjushkina O.Y. (2010). Prevention of cardiolipin oxidation and fatty acid cycling as two antioxidant mechanisms of cationic derivatives of plastoquinone (SkQs). Biochim. et Biophys. Acta (BBA) - Bioenerg..

[144] Skulachev V.P. (2013). Cationic antioxidants as a powerful tool against mitochondrial oxidative stress. Biochem. Biophys. Res. Commun..

[145] De Paepe R., Lemaire S.D., Danon A. (2014). Cardiolipin at the heart of stress response across kingdoms. Plant Signal. Behav..

